# Traumatic brain injury and the risk of dementia diagnosis: A nationwide cohort study

**DOI:** 10.1371/journal.pmed.1002496

**Published:** 2018-01-30

**Authors:** Anna Nordström, Peter Nordström

**Affiliations:** 1 Department of Public Health and Clinical Medicine, Environmental Medicine, Umeå University, Umeå, Sweden; 2 School of Sports Science, UiT The Arctic University of Norway, Tromsø, Norway; 3 Department of Community Medicine and Rehabilitation, Geriatric Medicine, Umeå University, Umeå, Sweden; University of Cambridge, UNITED KINGDOM

## Abstract

**Background:**

Traumatic brain injury (TBI) has been associated with dementia. The questions of whether the risk of dementia decreases over time after TBI, whether it is similar for different TBI types, and whether it is influenced by familial aggregation are not well studied.

**Methods and findings:**

The cohort considered for inclusion comprised all individuals in Sweden aged ≥50 years on December 31, 2005 (*n* = 3,329,360). Diagnoses of dementia and TBI were tracked through nationwide databases from 1964 until December 31, 2012. In a first cohort, individuals diagnosed with TBI (*n* = 164,334) were matched with up to two controls. A second cohort consisted of subjects diagnosed with dementia during follow-up (*n* = 136,233) matched with up to two controls. A third cohort consisted of 46,970 full sibling pairs with discordant TBI status. During a mean follow-up period of 15.3 (range, 0–49) years, 21,963 individuals in the first cohort (6.3% with TBI, 3.6% without TBI) were diagnosed with dementia (adjusted odds ratio [OR], 1.81; 95% confidence interval [CI], 1.75–1.86). The association was strongest in the first year after TBI (OR, 3.52; 95% CI, 3.23–3.84), but the risk remained significant >30 years (OR, 1.25; 95% CI, 1.11–1.41). Single mild TBI showed a weaker association with dementia (OR, 1.63; 95% CI, 1.57–1.70) than did more severe TBI (OR, 2.06; 95% CI, 1.95–2.19) and multiple TBIs (OR, 2.81; 95% CI, 2.51–3.15). These results were in general confirmed in the nested case-control cohort. TBI was also associated with an increased risk of dementia diagnosis in sibling pairs with discordant TBI status (OR, 1.89; 95% CI, 1.62–2.21). A main limitation of the present study is the observational design. Thus, no causal inferences can be made based on the associations found.

**Conclusions:**

The risk of dementia diagnosis decreased over time after TBI, but it was still evident >30 years after the trauma. The association was stronger for more severe TBI and multiple TBIs, and it persisted after adjustment for familial factors.

## Abstract 2

Using longitudinal, case-control, and sibling-matched analyses of nationwide data from Sweden, Peter Nordström and Anna Nordström describe the association between TBI and dementia, its time course, and the influence of familial factors.

## Author summary

**Why was this study done?:**

- Traumatic brain injury (TBI) is a leading cause of death and disability worldwide, especially in younger individuals. TBI has also been associated with the risk of dementia in older age.
- However, the results from previous studies are not conclusive, there is lack of studies with long follow-up, and no study has evaluated whether familial factors may influence the association.

**What did the researchers do and find?:**

- From all inhabitants living in Sweden above 50 years of age, three cohorts were formed. In the first cohort, 164,334 individuals with TBI were matched with controls; in the second case-control cohort, 136,233 individuals with dementia were matched with controls; and in the third cohort, 46,970 full sibling pairs discordant for TBI were evaluated for dementia during follow-up.
- In the retrospective cohort and case-control cohort, the risk of dementia was increased by four to six times the first year after TBI. Thereafter, the risk decreased rapidly but was still significant more than 30 years after the TBI.
- The risk of dementia was higher for those with a severe TBI or multiple TBIs, compared to those with one mild TBI.
- The results were generally confirmed in the sibling cohort.

**What do these findings mean?:**

- The risk of dementia is increased decades after a TBI; severe TBI or multiple TBIs increase the risk, compared to a mild TBI.
- Further studies are needed to evaluate whether the associations found are causal.

## Introduction

Traumatic brain injury (TBI) is a leading cause of death and disability in individuals aged <45 years in industrialized countries, and it is associated with developing a broad spectrum of pathophysiological symptoms, followed by long-term disability [1]. Accumulating evidence suggests that TBI is also associated with risk of developing dementia [2], a neurodegenerative disease with far-reaching social and medical implications.

Two meta-analyses of retrospective case-control studies have suggested that the risk of Alzheimer disease (AD) is doubled in men, but not in women, after TBI resulting in loss of consciousness [3,4]. Furthermore, a grading system for the risk of developing dementia based on TBI severity has been proposed for non-AD dementia [5] and for AD [6]. The retrospective MIRAGE study documented a fourfold increased risk of AD associated with TBI resulting in loss of consciousness and a twofold increased risk for TBI not resulting in loss of consciousness [6]. Other studies have yielded conflicting results, suggesting no association between previous TBI resulting in loss of consciousness and the development of AD or other types of dementia [7,8].

A previous study from our research group showed that the risk of developing young-onset dementia (YOD) after TBI was low for AD but strongly related to non-AD dementia, in a nationwide population-based cohort of 811,622 men and more than 30 years of follow-up [9]. Other researchers have suggested that a history of TBI accelerates the development of minimal cognitive impairment and AD; a retrospective study showed that the onset of dementia may occur ≥2 years earlier in individuals with TBI [10].

Thus, the details of how TBI is associated with the development, the time of onset, and the different types of dementia remain unclear. The aim of the present study was to examine whether different types of dementia diagnoses are associated with previous TBI and whether any observed association is time dependent, in a nationwide cohort.

## Materials and methods

### Materials

The cohort considered for inclusion in the present study included all men and women aged ≥50 years who lived in Sweden on December 31, 2005 (*n* = 3,329,360). Using data from Statistics Sweden (www.scb.se), information about early disability pension, civil status, and educational attainment in 2005 was linked to each individual in the cohort. From the total cohort, three component cohorts were formed. In the first retrospective cohort, individuals with TBI diagnoses and no prior diagnosis of dementia were each matched with two individuals without TBI during follow-up, based on birth year and sex. The baseline date for TBI cases and controls was the date of TBI. Controls who died or had a diagnosis of dementia prior to baseline were excluded. This procedure was repeated up to three times for each case. The National Patient Register was searched from 1964 through 2012, to identify prospective diagnoses of dementia.

The second cohort consisted of all full sibling pairs from the total cohort with discordant TBI status during follow-up. The baseline date for each pair was the date of TBI. Sibling pairs with death of the sibling without TBI or a diagnosis of dementia in at least one sibling before baseline were excluded. Prospective diagnoses of dementia from 1964 through 2012 were identified using the National Patient Register. The purpose of the sibling cohort was to adjust for potential uncontrolled confounding due to familial factors that would not be captured in the medical record.

**Fig 1:**
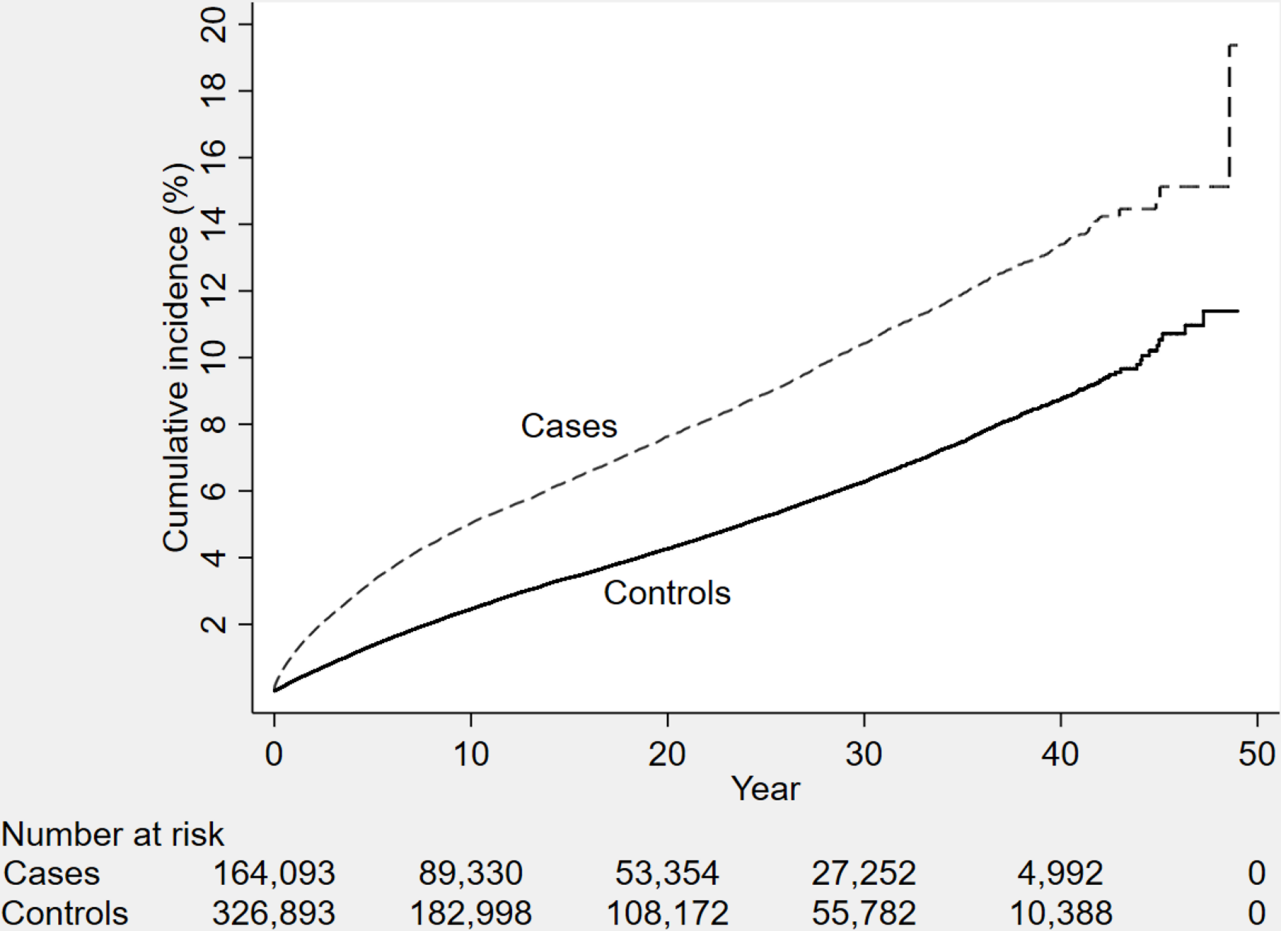
The cumulative incidence of dementia diagnosis during follow-up in individuals with and without TBI at baseline (*n* = 491,252 individuals). TBI, traumatic brain injury.

**Fig 2:**
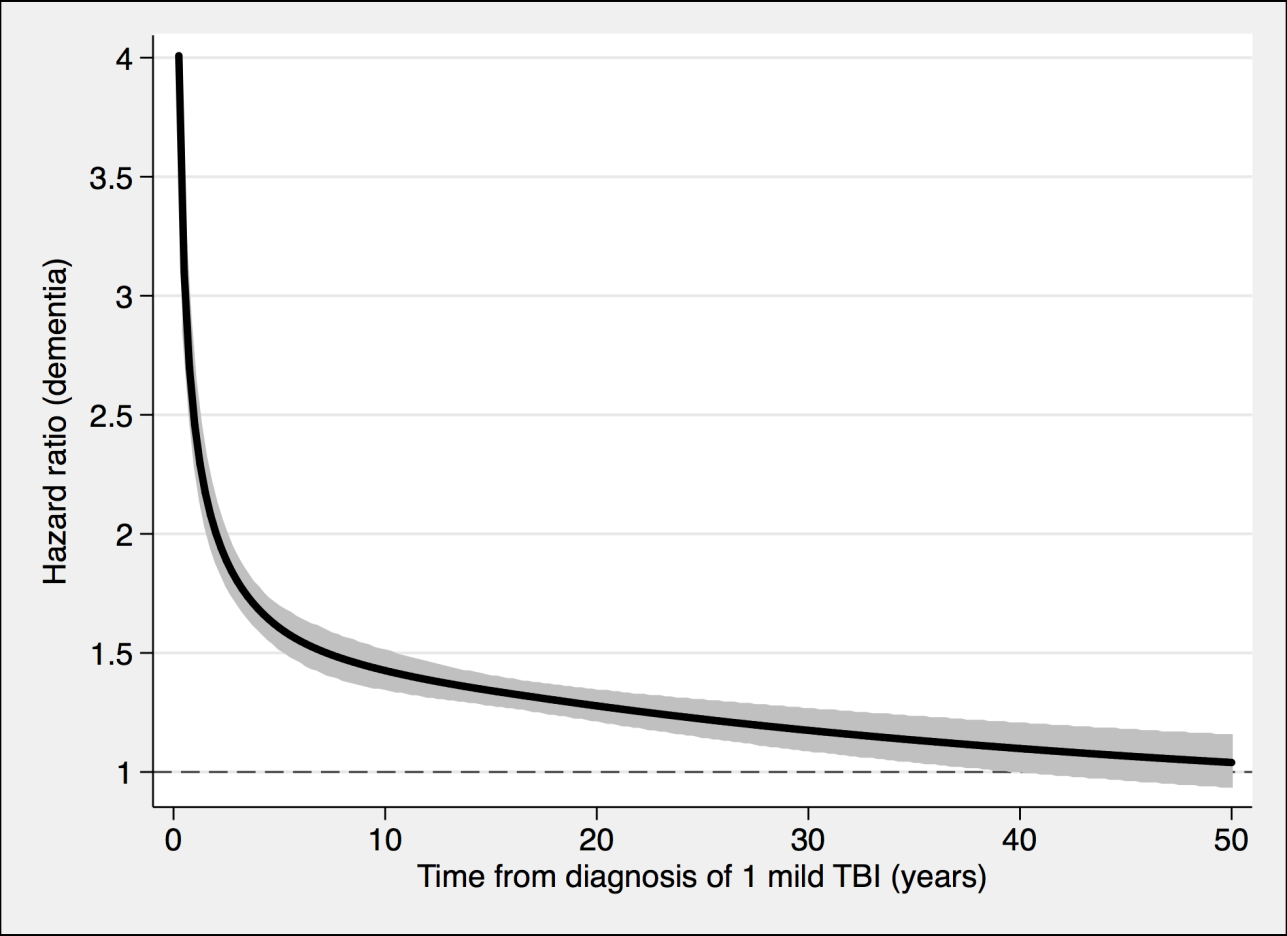
The risk of a dementia diagnosis in individuals with only one mild TBI (*n* = 108,463) and corresponding controls (*n* = 216,077), during follow-up. To model the effects, restricted cubic splines with four knots were used (resulting in three degrees of freedom), followed by fitting a proportional hazards model. TBI, traumatic brain injury.

**Fig 3:**
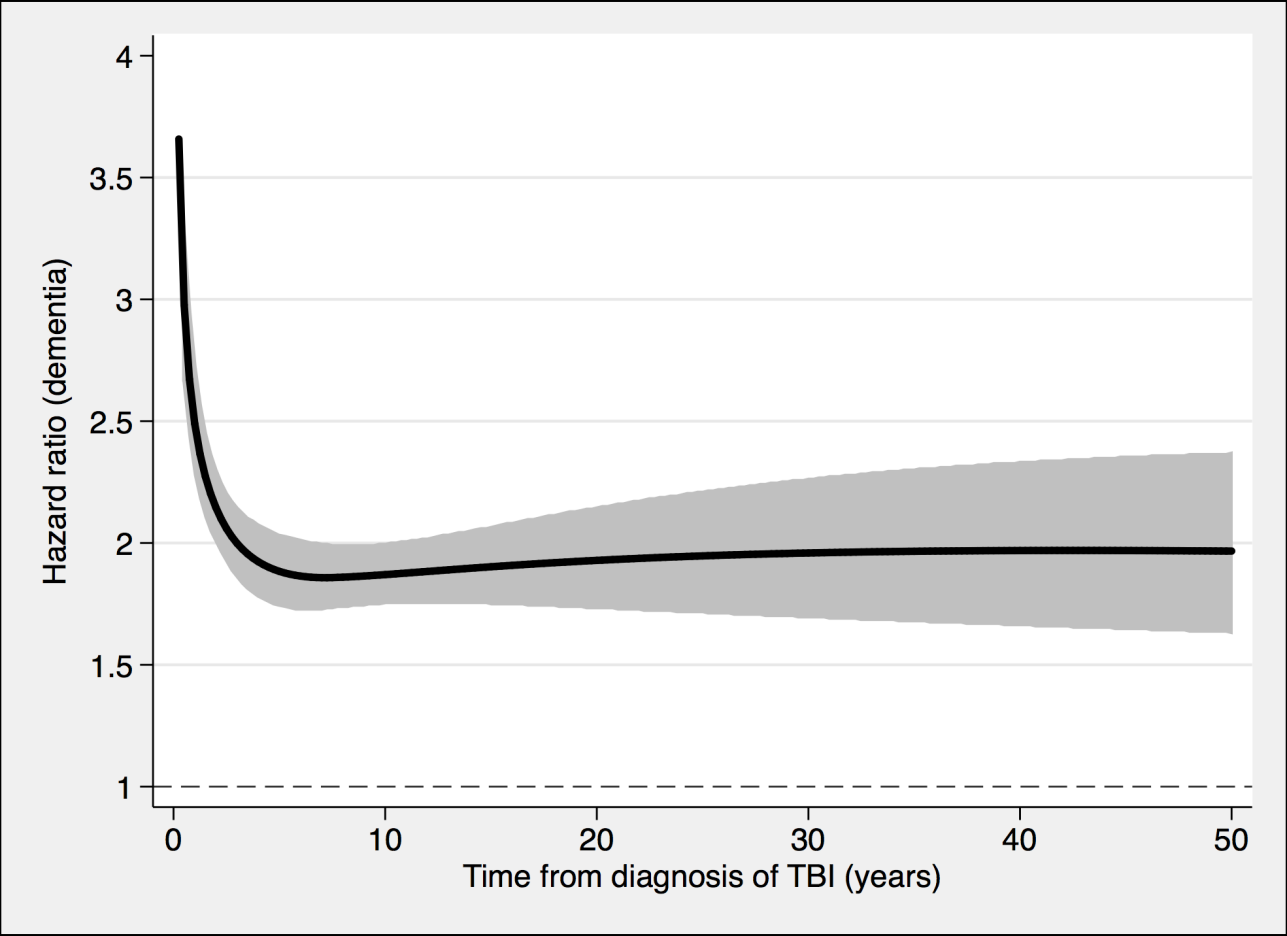
The risk of a dementia diagnosis in individuals with at least one nonmild TBI (*n* = 39,374) and corresponding controls (*n* = 77,924), during follow-up. To model the effects, restricted cubic splines with four knots were used (resulting in three degrees of freedom), followed by fitting a proportional hazards model. TBI, traumatic brain injury.

In the third cohort, all subjects diagnosed with dementia during follow-up were matched with up to two controls with no dementia diagnosis during follow-up, based on birth year and sex. Controls who died prior to the date of dementia for the corresponding cases were excluded. This procedure was repeated up to three times for each case. The baseline date in this case-control cohort was the date of dementia for each case and corresponding controls. In this cohort, a retrospective search of the National Patient Register through 1964 was conducted to identify TBIs occurring before baseline. The purpose of this nested case-control cohort was to evaluate the results from the cohort study. The Regional Ethical Review Board in Umeå and the National Board of Health and Welfare approved this study. There was no written prospective research protocol for the analyses presented in the present study. However, the analyses presented were preplanned, with the exception of Fig 1, Fig 2 and Fig 3, which were constructed during the revision process in response to reviewer comments.

### Diagnoses of dementia and TBI, death, and other covariates of interest

The Swedish National Patient Register (SNPR), controlled by the National Board of Health and Welfare, was searched through December 31, 2012, to identify diagnoses of dementia and TBI using appropriate International Classification of Disease (ICD; 8th, 9th, and 10th revisions) codes. Diagnoses of dementia were categorized as AD (ICD-10 code F00), vascular dementia (ICD-10 code F01), and dementia of unspecified type (ICD-10 code F039). For the diagnosis of dementia, the ICD-8 and ICD-9 code 290 was also included. TBI was coded as mild (ICD-10 code S060, ICD-8 and ICD-9 code 850) and more severe (ICD-10 code S06x, excluding S060, ICD-8 and ICD-9 code 851). A second TBI was defined as a new diagnosis recorded at least 6 months after the first diagnosis. Other diagnoses were selected based on known associations with the main exposure, outcome, or death; these included myocardial infarction, stroke, cancer, kidney failure, chronic pulmonary disease, atrial fibrillation, alcohol intoxication, depression, and diabetes. Diagnoses recorded in the SNPR have been validated, with positive predictive values of 85%–95% [11]. The SNPR has a national coverage rate of about 90% for inpatient care from 1970, and all specialized outpatient care has been included since 2001. Diagnoses of death were collected from the National Death Register, also controlled by the National Board of Health and Welfare.

### Statistical analysis

To test whether the association between TBI and the risk of subsequent dementia was time dependent in the prospective cohort, we evaluated Schoenfeld’s residuals using the estat phtest command in the Stata software (version 12.1; StataCorp LP, TX). As the test indicated that the proportional hazards assumption was violated, the association between TBI and the risk of dementia was analyzed in intervals of follow-up using multivariable adjusted conditional logistic regression in all three cohorts. For this purpose, the clogit command in the Stata software was used to fit maximum likelihood (fixed-effect) models with the dichotomous dependent variable of interest, i.e., dementia in the retrospective cohort study and sibling cohort, and TBI in the case-control study. The likelihood was then calculated relative to each group, i.e., conditional likelihood was used. The first model was unadjusted, although adjusted for age and sex by design. The second model was additionally adjusted for age at baseline, civil status, education, early retirement pension, and 10 diagnoses at baseline (Table 1). To further illustrate the nonlinear association over time in the cohort study and sibling cohort, restricted cubic splines with four knots were used (resulting in three degrees of freedom), followed by fitting a proportional hazards model [12]. The Stata software and SPSS (version 23; IBM, NY) were used to fit the statistical models and graphically illustrate the results.

**Table 1:** Cohort characteristics at baseline. The first (prospective) cohort was matched according to TBI at baseline, and the risk of dementia was investigated during follow-up; the second cohort consisted of siblings with discordant TBI at baseline; the third (retrospective) cohort was matched according to dementia during follow-up, and diagnoses of TBI were investigated.

Characteristic	Retrospective cohort study (*n* = 491,252)		Sibling cohort (*n* = 93,940)		Case-control cohort (*n* = 404,887)	
	TBI	No TBI	TBI	No TBI	Dementia	No dementia
**Age at baseline**	58.9 ± 18.7	58.7 ± 18.6	47.6 ± 15.7	47.7 ± 15.3	81.0 ± 3.3	80.8 ± 3.3
**Civil status**						
Married	73,290 (44.6%)	181,933 (55.7%)	23,355 (49.8%)	28,233 (60.1%)	56,434 (41.6%)	118,155 (43.9%)
Not married	25,175 (15.3%)	40,538 (12.4%)	9,374 (20.0%)	7,197 (15.3%)	10,910 (8.0%)	20,259 (7.5%)
Divorced	36,550 (22.2%)	48,376 (14.8%)	12,084 (25.7%)	9,186 (19.6%)	17,704 (13.0%)	27,511 (10.2%)
Widow/widower	29,247 (17.8%)	55,947 (17.1%)	2,122 (4.5%)	2,326 (5.0%)	50,640 (37.3%)	101,114 (37.6%)
**Education**						
Elementary school	67,518 (41.9%)	134,403 (41.8%)	14,656 (31.3%)	13,826 (29.5%)	74,752 (57.0%)	152,147 (58.6%)
2 y of sec. school	46,954 (29.1%)	85,646 (26.6%)	15,235 (32.5%)	15,105 (32.2%)	30,938 (23.6%)	57,745 (22.2%)
>2 y of sec. school	15,988 (9.9%)	33,932 (10.6%)	5,377 (11.5%)	5,494 (11.7%)	9,516 (7.3%)	17,656 (6.8%)
University education	30,848 (19.1%)	67,423 (21.0%)	11,570 (24.7%)	12,424 (26.5%)	15,925 (12.1%)	32,235 (12.4%)
**Early retirement pension**	28,449 (17.3%)	30,196 (9.2%)	12,073 (25.7%)	8,122 (17.3%)	5,057 (3.7%)	4,580 (1.7%)
**Diagnoses at baseline**						
Myocardial infarction	6,577 (4.0%)	10,956 (3.4%)	827 (1.8%)	559 (1.2%)	13,739 (10.1%)	24,242 (9.0%)
Ischemic stroke	7,556 (4.6%)	8,438 (2.6%)	871 (1.9%)	400 (0.9%)	19,138 (14.1%)	22,221 (8.3%)
Hemorrhagic stroke	4,493 (2.7%)	1,158 (0.4%)	791 (1.7%)	71 (0.2%)	3,606 (2.7%)	3,032 (1.1%)
Cancer	27,408 (20.2%)	55,157 (20.5%)	3,073 (6.5%)	2,920 (6.2%)	27,408 (20.2%)	55,157 (20.5%)
Atrial fibrillation	18,656 (13.7%)	28,494 (10.6%)	941 (2.0%)	465 (1.0%)	18,656 (13.7%)	28,494 (10.6%)
Chronic pulmonary disease	5,475 (4.0%)	8,069 (3.0%)	457 (1.0%)	276 (0.6%)	5,475 (4.0%)	8,069 (3.0%)
Renal failure	2,832 (2.1%)	3,713 (1.4%)	193 (0.4%)	97 (0.2%)	2,832 (2.1%)	3,713 (1.4%)
Alcohol intoxication	5,186 (3.8%)	3,525 (1.3%)	5,086 (10.8%)	1,025 (2.2%)	5,186 (3.8%)	3,525 (1.3%)
Diabetes	17,843 (13.1%)	23,873 (8.9%)	1,819 (3.9%)	1,029 (2.2%)	17,843 (13.1%)	23,873 (8.9%)
Depression	12,065 (8.9%)	6,936 (2.6%)	1,288 (2.7%)	516 (1.1%)	12,065 (8.9%)	6,936 (2.6%)

**Abbreviations:** sec., secondary; TBI, traumatic brain injury.

## Results

### Baseline characteristics

Characteristics of the retrospective cohort study and the sibling cohort, in which the risk of dementia diagnoses after baseline was investigated, and characteristics of the case-control cohort, in which the risk of TBI before baseline was investigated, are presented in Table 1. In the retrospective cohort study, more individuals with than without TBI were divorced, received early retirement pensions, and had diagnoses of diabetes and depression. Similar characteristics were found in the sibling cohort, although the mean age at baseline was lower and diagnoses were less common. In the case-control study, individuals with dementia more often had myocardial infarction, stroke, atrial fibrillation, depression, and diabetes at baseline than did those without dementia.

### Prospective risk of dementia diagnosis in the cohort study

**Fig 4:**
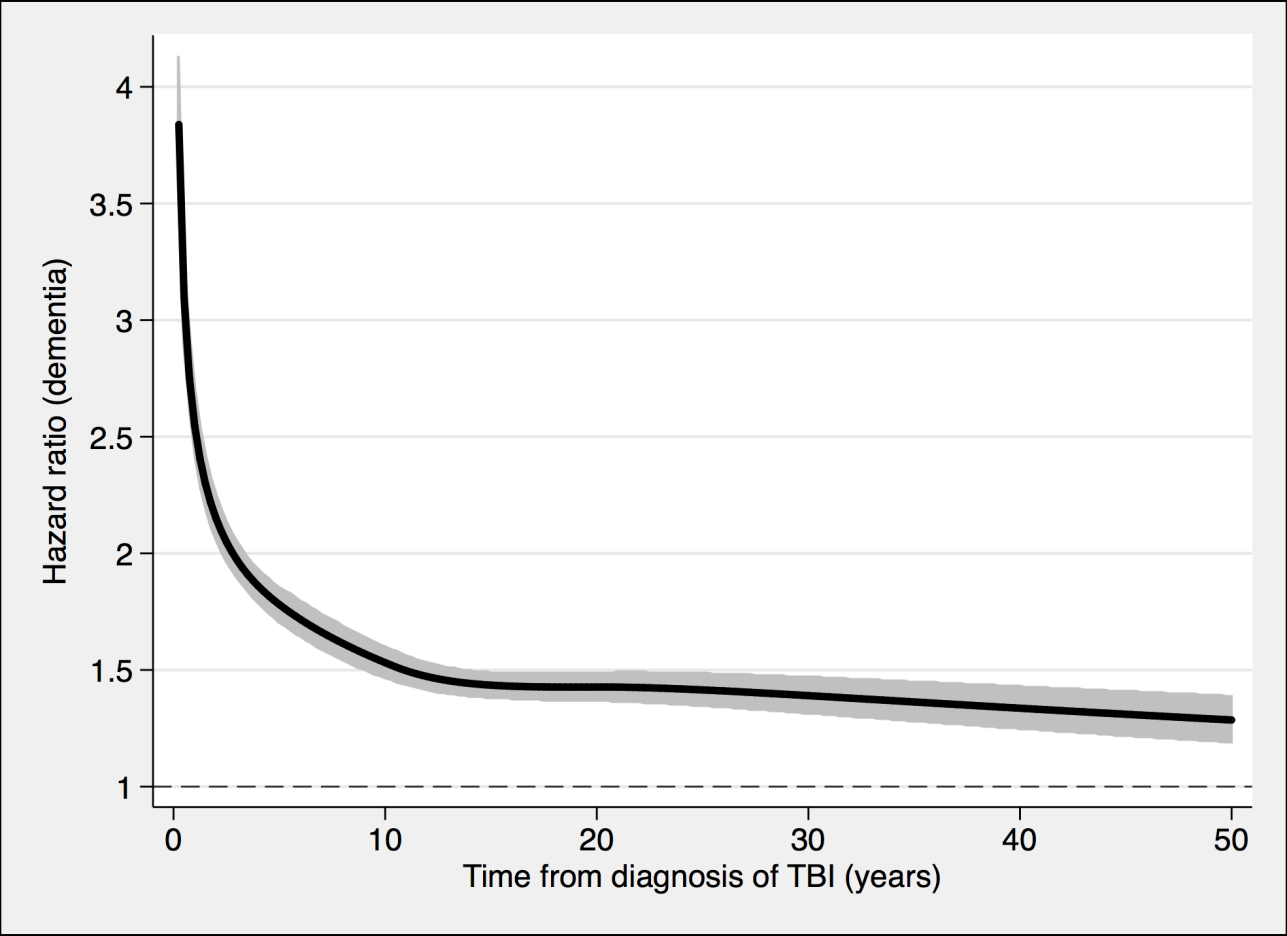
Associations between TBI and the risk of dementia diagnosis during follow-up in 491,252 individuals. To model the effects, restricted cubic splines with four knots were used (resulting in three degrees of freedom), followed by fitting a proportional hazards model. TBI, traumatic brain injury.

During a mean follow-up period of 15.3 (range, 0–49) years, 21,963 individuals in the total cohort (6.3% of those diagnosed with TBI, 3.6% of the rest of the cohort) were diagnosed with dementia (fully adjusted odds ratio [aOR], 1.81; 95% confidence interval [CI], 1.75–1.86; Table 2, Fig 4). Other strong risk factors at baseline (*p* < 0.001 for all) included higher age (aOR, 1.13), early retirement pension (aOR, 3.10), alcohol intoxication (aOR, 1.75), and depression (aOR, 1.41). The association was similar in men (aOR, 1.88; 95% CI, 1.80–1.97) and in women (aOR, 1.75; 95% CI, 1.68–1.82). The risk of dementia diagnosis after TBI decreased rapidly the first year (Fig 4). Thus, the association between TBI and subsequent dementia was strongest in the first year after TBI (aOR, 3.52; 95% CI, 3.23–3.84; Table 2) but still increased more than 30 years after TBI (aOR, 1.25; 95% CI, 1.11–1.41; Table 2). The association between TBI and dementia was weaker for the outcome of AD (aOR, 1.58; 95% CI, 1.49–1.69) than for vascular dementia (aOR, 2.17; 95% CI, 2.02–2.32) and unspecified dementia (aOR, 1.78; 95% CI, 1.71–1.85). The risk of dementia diagnosis associated with one mild TBI and one more severe TBI both decreased rapidly the first years after TBI (Fig 2 and Fig 3, respectively). Overall, single mild TBI showed the weakest association with dementia (aOR, 1.63; 95% CI, 1.57–1.70), with stronger associations for more severe TBI (aOR, 2.06; 95% CI, 1.95–2.19) and multiple TBIs (aOR, 2.81; 95% CI, 2.51–3.15). The association between dementia and TBI was stronger for those diagnosed with dementia before the age of 65 years (aOR, 2.25; 95% CI, 1.91–2.65) than for those diagnosed after this age (aOR, 1.79; 95% CI, 1.74–1.85).

**Table 2:** Associations between TBI and the risk of dementia during follow-up in 491,252 individuals. Odds ratios and 95% CIs were derived from conditional logistic regression analysis.

Period after the TBI	Individuals at risk	Diagnosed with dementia	Unadjusted		Adjusted*	
			Odds ratio	95% CI	Odds ratio	95% CI
Overall	491,252	21,963	1.90	1.85–1.96	1.81	1.75–1.86
1–364 days	491,252	2,784 (0.6%)	3.69	3.40–3.99	3.52	3.23–3.84
1–4.9 years	458,525	5,893 (1.3%)	2.37	2.24–2.51	2.24	2.11–2.38
5–9.9 years	362,201	4,234 (1.2%)	1.86	1.74–1.99	1.75	1.63–1.88
10–19.9 years	272,328	4,644 (1.7%)	1.58	1.48–1.68	1.48	1.39–1.58
20–29.9 years	161,526	2,916 (1.8%)	1.50	1.39–1.63	1.40	1.29–1.52
30 years or more	83,034	1,492 (1.8%)	1.28	1.15–1.44	1.25	1.11–1.41

*Adjusted for age, civil status, education, early retirement pension, and diagnoses at baseline.

**Abbreviations:** CI, confidence interval; TBI, traumatic brain injury.

### Prospective risk of dementia diagnosis in the sibling cohort

**Fig 5:**
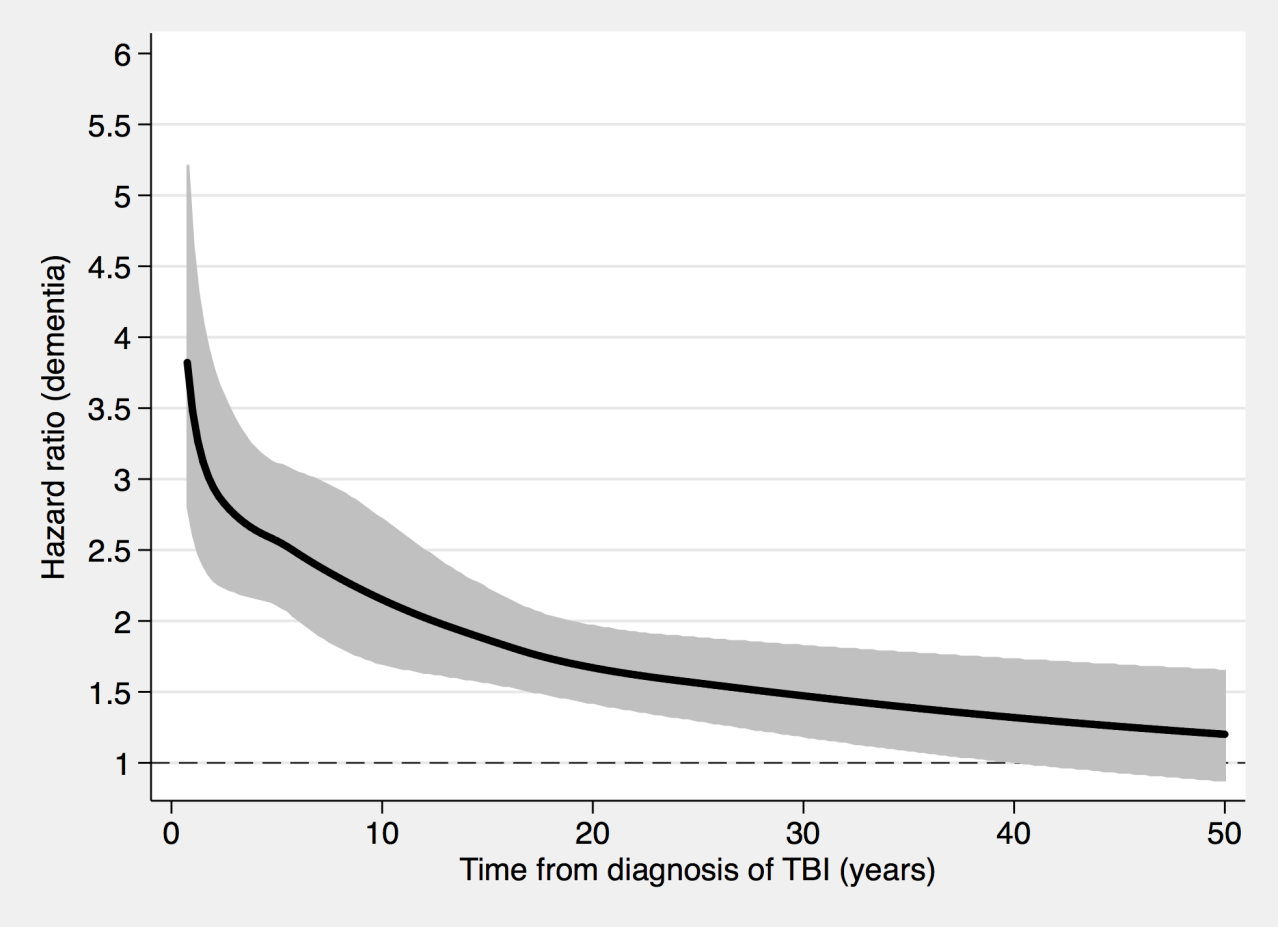
Associations between TBI and a dementia diagnosis during follow-up in 46,970 sibling pairs discordant for TBI. To model the effects, restricted cubic splines with four knots were used (resulting in three degrees of freedom), followed by fitting a proportional hazards model. TBI, traumatic brain injury.

During a mean follow-up period of 18.8 (range, 0–49) years, 1,204 individuals in the sibling cohort (1.8% of siblings with TBI at baseline, 0.8% of unaffected siblings) were diagnosed with dementia (aOR, 1.89; 95% CI, 1.62–2.21; Table 3 and Fig 5). The association between TBI and the risk of subsequent dementia was similar to that in the total cohort not consisting of siblings (Table 2). In fully adjusted models, the risk of dementia was highest in the first year after TBI (aOR, 6.09; 95% CI, 2.70–13.71; Table 3); it declined gradually but remained significant more than 10 years after TBI (aOR, 1.39; 95% CI, 1.12–1.72). The association was weaker for single mild TBI (aOR, 1.49; 95% CI, 1.23–1.80) than for single severe TBI (aOR, 3.31; 95% CI, 2.13–5.16) and multiple TBIs (aOR, 5.01; 95% CI, 2.37–10.61).

**Table 3:** Associations between TBI and the risk of dementia during follow-up in 46,970 full sibling pairs, discordant for TBI at baseline. Odds ratios and 95% CIs were derived from conditional logistic regression analysis.

Period after the TBI	Individuals at risk	Diagnosed with dementia	Unadjusted		Adjusted*	
			Odds ratio	95% CI	Odds ratio	95% CI
Overall	93,940	1,204	2.32	2.05–2.63	1.89	1.62–2.22
1–364 days	93,940	123 (0.1%)	5.15	3.19–8.31	6.09	2.70–13.71
1–4.9 years	89,709	311 (0.3%)	3.41	2.59–4.50	2.83	2.00–4.00
5–9.9 years	75,868	204 (0.3%)	2.65	1.93–3.65	2.05	1.29–3.27
10 years or more	60,723	566 (0.6%)	1.67	1.40–1.99	1.39	1.12–1.72

*Adjusted for age, civil status, education, early retirement pension, and diagnoses at baseline.

**Abbreviations:** CI, confidence interval; TBI, traumatic brain injury.

### Retrospective risk of TBI diagnosis in the case-control cohort

In total, 21,276 individuals in this cohort (7.7% of individuals with dementia, 4.0% of the rest of the cohort; *p* < 0.001) had a history of at least one TBI before baseline. After adjustment for all confounders, the risk of dementia was highest in the first year after TBI (OR, 3.89; 95% CI, 3.54–4.27; Table 4). This elevated risk was still significant 30 years after TBI in the unadjusted analysis (OR, 1.20; 95% CI, 1.09–1.32) but not after adjustment for all confounders (OR, 1.08; 95% CI, 0.97–1.19; Table 4). Overall, single mild TBI showed the weakest association with dementia (aOR, 1.59; 95% CI, 1.54–1.65), with stronger associations for more severe TBI (aOR, 1.95; 95% CI, 1.83–2.08) and multiple TBIs (aOR, 2.15; 95% CI, 1.93–2.40). The association was similar for diagnoses recorded before the age of 65 years (aOR, 1.75; 95% CI, 1.47–2.08) and after this age (aOR, 1.71; 95% CI, 1.65–1.76).

**Table 4:** Associations between dementia at baseline and previous TBI in the retrospective cohort (*n* = 404,887). Odds ratios and 95% CIs were derived from conditional logistic regression analysis.

Period before dementia	Individuals at risk	Diagnosed with TBI	Unadjusted		Adjusted*	
			Odds ratio	95% CI	Odds ratio	95% CI
Overall	404,887	21,276	1.99	1.93–2.04	1.71	1.66–1.76
1–364 days	386,358	2,747 (0.7%)	4.34	4.09–4.77	3.89	3.54–4.27
1–4.9 years	391,831	5,473 (1.4%)	2.40	2.27–2.54	2.07	1.95–2.20
5–9.9 years	395,813	3,982 (1.0%)	1.80	1.69–1.91	1.54	1.43–1.65
10–19.9 years	400,213	4,400 (1.1%)	1.49	1.41–1.59	1.30	1.22–1.39
20–29.9 years	403,107	2,894 (0.7%)	1.35	1.25–1.45	1.18	1.08–1.28
30 years of more	404,887	1,780 (0.4%)	1.20	1.09–1.32	1.08	0.97–1.19

*Adjusted for age, civil status, education, early retirement pension, and diagnoses at baseline.

**Abbreviations:** CI, confidence interval; TBI, traumatic brain injury.

## Discussion

In the present nationwide cohort, with up to 50 years of follow-up, a clear association was observed between previous TBI and the risk of being diagnosed with dementia later in life. The risk of dementia was highest in the first years after TBI, but it was sustained more than 30 years thereafter. The association was also similar in a large cohort of full siblings and similar in men and in women. Finally, the risk of developing dementia appeared to have a dose–response relationship with regard to TBI severity and number of TBIs.

The link between TBI and developing dementia has been controversial for some time, due to the conflicting nature of available data. In the present study, the risk of dementia diagnosis was increased by about 80% during a mean follow-up period of 15 years for individuals diagnosed with TBI, compared with the rest of the cohort. The investigation of TBI as a risk factor for dementia entails the risk of reversed causality [13–15] or misdiagnosis due to post-concussive symptoms; thus, data from studies with short follow-up periods [16] should be interpreted with caution. In the aging population, dementia can be an underlying risk factor for accidents resulting in TBI, such as car accidents and injurious falls [17,18]. In the present study, the risk of being diagnosed with dementia in the first year after TBI was four to six times higher, compared with individuals with no TBI. Thereafter, this risk declined rapidly. The development of dementia, with impaired executive function and an increased risk of falling, likely began before the time of TBI in some individuals in these cohorts; thus, TBI may have been influenced by reduced cognitive function, with resulting reversed causality. Nevertheless, the significant association observed more than 30 years after TBI cannot be explained by reversed causality. Still, an unknown confounder may explain the increased risk of dementia also with longer follow-up. In a previous study, the strength of the association between YOD and TBI was reduced markedly after adjustment for confounders [9]. As in the present study, a previous TBI showed stronger associations with non-AD forms. To our knowledge, no previous prospective study with similar power and follow-up time has been reported, preventing direct comparisons to our data.

The relation of TBI severity to the risk of developing dementia is a matter of much debate. Evidence from previous studies is not conclusive; several studies have suggested that moderate to severe TBI is an important risk factor for subsequent dementia [4,6,19–21], but others have failed to confirm these results [14,22,23]. The lack of association could be due to limited statistical power, as severe and multiple TBIs are less common than single mild TBI, including in the present study. Data from the present study suggest a clear dose–response relationship, with single mild TBI showing a weaker association with dementia diagnosis than did more severe and multiple TBIs. In support, a recent nationwide Finnish study found that persons with more severe TBI were at increased risk of dementia, compared to those with mild TBI [24]. These graded associations may support the existence of a causal relationship. Another explanation could be that subjects with more severe or multiple TBIs more often have other risk factors for dementia, such as lower cognitive function before TBI [25].

The associations found between TBI and the risk of subsequent dementia diagnosis could also be influenced by familial factors, such as upbringing conditions, education, and genetic factors. To our knowledge, no previous population-based study with a long follow-up period has evaluated these potential influences. In the present study, we thus examined the association in about 47,000 full sibling pairs with discordant TBI status during follow-up. The risk of dementia diagnosis during follow-up was almost doubled in siblings with TBI compared with their counterparts without TBI, and it remained increased more than 10 years after TBI. These results are similar to those obtained for the other cohorts, suggesting that familial factors cannot explain the association between TBI and dementia.

The present study has several limitations that should be considered. Most importantly, no causal inference should be made based on observational data, although the time-dependent associations more than 30 years after TBI and the dose-dependent relationship according to TBI severity and number may support such causality. The strong association demonstrated for individuals with short follow-up is most likely subject to different forms of bias, e.g., reversed causality, as discussed previously. In addition, it is likely that individuals with TBI are subject to a more rigorous control from healthcare and relatives initially after TBI, increasing the chance of being diagnosed with dementia. The results of the present study are based on diagnoses made in specialist care; diagnoses made in primary care were not included, which may have affected the numbers of TBI and dementia diagnoses included in analyses. Furthermore, data were obtained from registers and diagnoses could not be confirmed clinically, although all diagnoses were recorded in the context of specialist healthcare. Nevertheless, a lower sensitivity with respect to these outcomes would, if anything, attenuate the associations found. The main strengths of the present study include the large body of recorded data covering a long period, which provided superior statistical power for the performance of reliable analyses, and a long follow-up period that was not subject to recall bias. The main results of the present study were also evaluated in a cohort of siblings, and the findings were consistent. Thus, this sensitivity analysis supported the validity of the main findings, and because the cohort was nationwide, the external validity of the results is likely to be high.

In summary, the findings of this study suggest the existence of a time- and dose-dependent risk of developing dementia more than 30 years after TBI. The association was stronger for more severe and multiple TBIs than for single mild TBI. The association was also present after adjustment for familial factors in a sibling analysis. Overall, the results may support a causal association between TBI and the risks of different types of dementia. However, given the observational study design, we cannot exclude the possibility that other factors explain the observed associations.

## Supporting information

S1 STROBE Checklist (DOC)

## Abbreviations

AD: Alzheimer disease
aOR: fully adjusted odds ratio
CI: confidence interval
ICD: International Classification of Disease
OR: adjusted odds ratio
SNPR: Swedish National Patient Register
TBI: traumatic brain injury
YOD: young-onset dementia
